# Association between household solid fuel use and cognitive frailty in a middle-aged and older Chinese population

**DOI:** 10.3389/fpubh.2025.1444421

**Published:** 2025-03-26

**Authors:** Mo-Yao Tan, Gao-Peng Wang, Si-Xuan Zhu, Li-Hai Jiang

**Affiliations:** ^1^Chengdu Integrated TCM and Western Medicine Hospital, Chengdu, Sichuan, China; ^2^Chengdu University of Traditional Chinese Medicine, Chengdu, Sichuan, China

**Keywords:** household solid fuel, cognitive frailty, cohort study, CHARLS, Chinese older adult individuals

## Abstract

**Objectives:**

Our research intended to investigate the association between the solid fuels use and the risk of cognitive frailty (CF).

**Methods:**

The research utilized data from the China Health and Retirement Longitudinal Study (CHARLS), a nationwide longitudinal study focusing on individuals aged 45 and older. A total of 8,563 participants without CF were enrolled from 2011 and followed up to 2015. Household fuel types include solid fuels (such as coal, crop residue, or wood-burning) and clean fuels (such as solar power, natural gas, liquefied petroleum gas, electricity, or marsh gas). CF was defined as the co-existence of cognitive impairment and physical frailty. Cox proportional hazards models were utilized to evaluate the relationship between the solid fuels use and the risk of CF. Furthermore, sensitivity analyses were conducted.

**Results:**

Over a median follow-up of 4.0 years, 131 subjects were diagnosed with CF. We observed that the solid fuels use for cooking or heating increased the risk of developing CF compared to clean fuels, with HRs of 2.02 (95% CI: 1.25 to 3.25) and 2.38 (95% CI: 1.26 to 4.48), respectively. In addition, participants who use solid fuel for heating (HR: 2.38 [95% CI: 1.26, 4.48]) and cooking (HR: 2.02 [95% CI: 1.25, 3.25]) might experience an increased risk of CF. However, transitioning from solid to clean fuels for cooking could potentially reduce these risks (HR: 0.38 [95% CI: 0.16, 0.88]).

**Conclusion:**

Household solid fuels utilization was closely associated with the risk of CF.

## Introduction

Cognitive frailty (CF) is a complex condition that gradually develops with aging (1). Previous studies have shown that cognitive impairment (CI) is significantly associated with physical frailty (PF) in older adults, as the two conditions frequently coexist (2, 3). In response to this, a consensus meeting held by members of the International Academy on Nutrition and Aging (I.A.N.A.) and the International Association of Gerontology and Geriatrics (I.A.G.G.) recently established the concept of CF (1). CF was defined as the coexistence of PF and mild CI without dementia. Global survey data indicate that the prevalence of CF among older adults in communities ranges from 1 to 5% (4), potentially affecting approximately 3.9 million older adult individuals in China (5). CF has a significant negative impact on health and is closely associated with an increased risk of disability, higher mortality rates, and a reduced quality of life (6).

PF, a key component of CF, has a global prevalence of approximately 12% and is primarily characterized by reduced walking speed and decreased muscle strength (7, 8). The impact of PF on cognitive functions is also noteworthy. Research has shown that low physical activity may further diminish cognitive reserves, triggering clinical or pathological manifestations of CI (9). Moreover, functional limitations in daily activities due to PF are closely linked to an increased risk of CI (10). CI involves declines in various cognitive domains, such as memory, visuospatial abilities, orientation, calculation, executive function, and comprehension (11). It is important to note that PF and CI frequently interact and coexist within the same individuals (12). The more holistic concept of CF integrates PF with CI, emphasizing the need for integrated healthcare strategies that address both cognitive and physical health challenges simultaneously (12, 13).

Globally, approximately 2.4 billion people depend on solid fuels, such as coal, crop residue, and wood burning, primarily for cooking and heating, which positions it as a leading cause of household air pollution (HAP) (14). Each year, over 3 million people worldwide die prematurely due to HAP (15). In typical developing countries like China, about 33% of the population remains reliant on solid fuels due to difficulties in accessing cleaner alternatives, particularly among lower-income and less-educated groups (16). The combustion of these solid fuels generates a significant amount of pollutants, including fine particulate matter (PM_2.5_ and PM_10_), carbon monoxide (CO), nitrogen dioxide (NO_2_), black carbon, and various organic compounds that are carcinogenic (17). These harmful substances pose a serious threat to human health, potentially causing cardiovascular and respiratory diseases, severe depressive symptoms, and cognitive function in the nervous system (18). Given the widespread use of solid fuels in households and the increasing recognition of CF, there is a lack of literature explicitly linking these two variables. Therefore, it is crucial to investigate the connection between utilization of solid fuels and its impact on CF.

In light of the current dearth of research pertaining to the link between domestic use of solid fuels and CF, and considering China’s evolution towards a population with a higher average age, we seek to explore if the utilization of indoor solid fuels among Chinese individuals aged 45 and older is linked to the likelihood of CF.

## Methods

### Study design and population

This study employed data from the China Health and Retirement Longitudinal Study (CHARLS), a comprehensive nationwide population cohort study. The research carried out within the framework of CHARLS has obtained ethical approval from the Biomedical Ethics Review Committee of Peking University (IRB 00001052–11,015), and all participants have provided their informed consent by signing consent forms. CHARLS, which was initiated in 2011 and is conducted every 2 years, has the primary objective of gathering nationally representative data from Chinese individuals aged 45 and above in order to advance gerontological research. This comprehensive study employs a meticulously designed multistage stratified probability proportional to size sampling method, which involves randomly selecting around 10,000 households across 450 villages/communities in 150 counties/districts throughout 28 provinces (19). Every participant is subjected to evaluation using standardized questionnaires, facilitating the methodical gathering of extensive data on sociodemographic characteristics, current indoor fuel exposures, lifestyle habits, and health-related information. The structured survey includes eight sections, with complete details accessible on the official CHARLS website.^1^ In 2014, the CHARLS introduced a pioneering life course survey aiming to reconstruct the life histories of Chinese residents aged 45 and above by delving into their past experiences. This particular survey offers detailed insights into the previous indoor fuel usage among participants of the 2011 baseline survey. To date, comprehensive data has been available from several phases: the initial baseline survey in 2011, followed by two-year (2013), four-year (2015), seven-year (2018), and nine-year (2020) follow-ups, along with the vital data from the 2014 life course survey.

**Figure 1:**
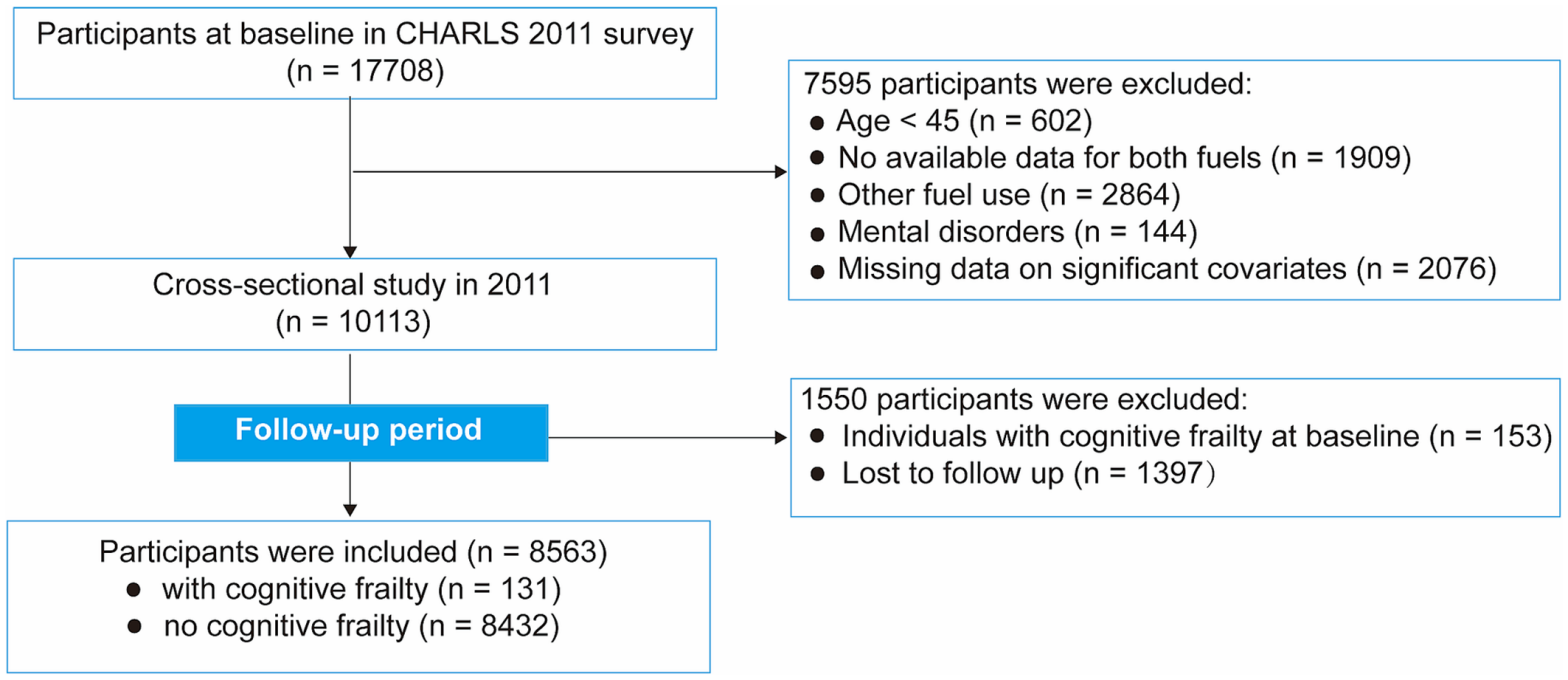
Flow chart of the study participant selection process.

Notably, the definition of CF encompasses a range of biochemical indicators, including body mass index (BMI), the chair stand test, 2.5-meter gait speed, and the grip strength test. Concerning biochemical data collection, the CHARLS was only conducted at critical intervals: initially at baseline in 2011, then in a second wave in 2013, and a third wave in 2015. Consequently, our analysis focused on the data collected within the 2011 to 2015 period. The preliminary phase of our study involved screening 17,708 individuals from the baseline survey conducted between 2011 and 2012. The criteria for excluding participants from the further analysis were as follows: those under the age of 45 (*n* = 602), missing data on both fuel use (*n* = 1909), use of other fuel types not specified (*n* = 2,864), diagnosis of mental disorders (*n* = 144), and missing information on significant covariates (*n* = 2076). Additionally, 151 participants who had prevalent CF at baseline and 1,397 participants who were lost to follow-up were also excluded. Ultimately, 8,563 participants were deemed eligible for our study, as illustrated in Figure 1.

### Definition of household solid fuel

Adapting the research methodology from Chen et al. (20), we employed two pivotal questionnaires to ascertain the predominant energy sources in households, including queries like “What is the main source of cooking fuel?” and “What is the primary heating energy source?.” Solid fuels were self-reported and categorized into coal, crop residues, or wood-burning used for cooking and heating purposes. In contrast, clean fuels encompassed the habitual use of solar energy, natural gas, liquefied petroleum gas, or electricity to meet cooking and heating demands. To ensure the precision and relevance of our study, we excluded responses indicating the use of “other” fuels (21). Fuel switching is assessed when a disparity in fuel utilization exists between the life course survey and the baseline (22). For instance, if the life course survey unveils an individual’s reliance on solid fuels, while the baseline indicates the adoption of clean fuels, this circumstance would be interpreted as a shift in the participant’s fuel use from clean fuels to solid fuels.

### Definition of PF

PF was conducted through an adapted version of the Fried phenotyping approach (23). This refined approach was adapted and validated within the CHARLS framework (24). As described previously (12, 24–26), PF encompasses five components: shrinking, weakness, slowness, low physical activity, and exhaustion. Shrinking was defined as having a BMI not exceeding 18.5 kg/m^2^ or self-reporting a weight loss of 5 kg or more in the preceding year. Weakness was evaluated by measuring handgrip strength with a dynamometer for both dominant and non-dominant hands, where participants were encouraged to exert their maximum effort. The criterion for defining weakness was set at a grip strength at or below the 20th percentile, taking into account adjustments for sex and BMI. Slowness was assessed through a 2.5-meter gait speed or the chair stand test, as outlined by Wu et al. (24). Participants were classified as having low physical activity based on their responses to three specific questions regarding their engagement in vigorous activities, moderate physical effort, and walking for at least 10 min continuously during a typical week (25). Negative responses to these questions resulted in their categorization into the low physical activity group.

The level of exhaustion was evaluated using two specific questions from the 10-item Center for Epidemiological Studies Depression Scale (CESD-10): “I felt everything I did was an effort” and “I could not get going.” Participants who responded with “sometimes or half of the time (3–4 days)” or “most of the time (5–7 days)” to either of these questions were categorized as experiencing self-reported exhaustion (25). Participants meeting three or more of the specified criteria were defined as PF.

### Definition of CI

Within the CHARLS, the evaluation of CI concentrated on two primary cognitive measures: episodic memory and executive function (27).

#### Episodic memory assessment

This is measured through immediate and delayed recall tests. The procedure involves reading 10 unrelated Chinese words to the participants, followed by an evaluation of their ability to recall these words both immediately and after a four-minute interval. The episodic memory score is calculated as the average of the immediate and delayed recall scores, with a range from 0 to 10.

#### Executive function assessment

This is based on the Telephone Interview for Cognitive Status (TICS) and a drawing test. The TICS involves tasks like recognizing the date (year, month, season, and day), the day of the week, and performing serial subtraction of 7 from 100 (5 times). In the drawing test, participants are asked to replicate a given image. The executive function score is the combined total of the TICS and drawing test scores, with a scale from 0 to 11.

Ultimately, the total cognitive function score is derived from adding the scores of episodic memory and executive function, with a maximum of 21 points. According to the literature, a total score below 6 is defined as indicative of CI, whereas a score above this threshold is considered indicative of normal cognitive function (27).

### Definition of CF

In accordance with the definition established by the International Consensus Group (I.A.N.A/I.A.G.G) (1), and as substantiated in the literature of preceding studies (5, 12, 25), the concept of CF is identified as the concurrent presence of CI and PF.

### Covariates

Based on prior research (20, 28), we included the following covariates: social activities, age (years), sex (female/male), education level (junior high school or below/senior high school or above), marital status (married/divorced/widowed/unmarried), self-reported socioeconomic status (poor/fair/good), residence (urban/rural), smoking status (non-smoker/current or former smoker), drinking status (non-drinker/current or former drinker), and the presence of hypertension, diabetes, and stroke (yes/no for each). Socioeconomic status was self-reported through the question, “How would you rate your standard of living?” with options ranging from poor to very high. Based on their responses, we classified them into three categories: “poor” (including relatively poor and poor), “fair” (equivalent to a fair standard of living), and “good” (comprising both relatively high and very high). The criteria for identifying hypertension were (29): (1) a systolic blood pressure of 140 mmHg or more, (2) a diastolic blood pressure of 90 mmHg or more, (3) a self-reported history of hypertension, or (4) the use of medication to lower blood pressure. Participants were deemed to have diabetes if they reported a diagnosis confirmed by a doctor, had fasting blood glucose levels of 126 mg/dL (7.0 mmol/L) or above, had glycosylated hemoglobin levels of 6.5% or above, or were taking medications for diabetes (30).

### Statistical analyses

Based on the cooking and heating fuel type, we presented baseline characteristic data with continuous variables expressed as mean ± standard deviation (SD) and categorical variables as percentages. We calculated CF incidence rates by individual cooking or heating fuel type, expressing these with 95% confidence intervals (CIs) as events per 1,000 person-years. The Cox proportional hazards model is a statistical methods employed to analyze the relationship between one or more explanatory variables (also known as covariates) and the hazard rate of a specific event (31). In the present study, we used Cox regression to evaluate the association between the solid fuels use and the hazard risk of CF. We assessed the proportional hazards models using Schoenfeld residuals and found no violations (32).

We first analyzed the different impacts of solid fuels for heating and cooking on the risk of CF. We then investigated the collective effects of fuel combinations on the development of CF ([reference group]: both clean fuels; [exposure group]: clean fuel use for cooking and solid fuel use for heating; solid fuel use for cooking and clean fuel use for heating; solid fuel use for both cooking and heating), including transitions from solid to clean fuels and vice versa. For each analysis, we calculated hazard ratios (HRs) and their 95% CIs. Participants were followed up from baseline to CF diagnosis, death, or the end of the follow-up, whichever came first. In our study, three Cox models were constructed: Model 1 was unadjusted, Model 2 adjusted for age and sex, and Model 3 with full adjustments including age, sex, education level, marital status, smoking, drinking status, residence, self-reported socioeconomic status, social activities, hypertension, diabetes, and stroke.

We conducted a comprehensive series of sensitivity analyses to rigorously assess and confirm the robustness of our findings. (1) Firstly, considering the diversity of solid fuel used among households in the CHARLS, we separately evaluated the individual impacts of coal use and crop residue/wood burning on the risk of developing CF. (2) Moreover, the published literature reveals variations in the definition of clean fuels for heating (33). These definitions can be broadly divided into two categories: solar energy, natural gas, liquefied petroleum gas, and electricity, whereas the other includes centralized heating alongside these previously mentioned fuels. In our sensitivity analysis, we specifically considered centralized heating as a factor within clean heating fuels. (3) To minimizing the influence of confounding factors and to uncover potential modification or interaction effects of stratified variables, as well as population-specific findings, we conducted stratified analyses based on age (≤65/>65), sex (female/male), socioeconomic status (poor/fair/good), drinking (nondrinker/ Current or former drinker), and smoking (nonsmoker/ current or former smoker). The interactions between these stratified covariates and CF were estimated using the likelihood ratio test. It is noteworthy that due to the uneven distribution of disease prevalence among the baseline population, we did not conduct stratified analyses for conditions such as hypertension, diabetes, and stroke.

A statistically significant result was determined when the two-tailed *p* value was less than 0.05. The statistical analyses were conducted utilizing the R software (version 4.1.2).

## Results

### Participant’s characteristics

Table 1 presents the fundamental characteristics of the included population categorized by the various types of household fuel utilization. Among the 10,232 study participants, we documented 131 incident CF, with females comprising 53.60% and males 46.40%, during a median observation period of 4.0 years. Regarding fuel usage, 62.60% of participants reported regularly using solid fuels for cooking, and 76.50% used solid fuels for heating; conversely, 37.40 and 23.50% of participants, respectively, used clean fuels for cooking and heating. Compared with participants who used clean fuels for heating or cooking, the group using solid fuels generally reported lower socioeconomic status and a higher proportion of current or former smokers and stroke patients. Furthermore, those using solid fuels for cooking or heating were often older, more likely to be unmarried or widowed, and tended to hold a junior high school diploma or lower, with a relatively higher risk of CF. Notably, urban dwellers often opted for clean fuels for heating or cooking purposes, whereas individuals in rural regions predominantly used solid fuels for these daily needs.

**Table 1:** Participant characteristics by household fuel type at baseline.

Characteristic	Household heating fuel			Household cooking fuel		
	Total	Clean fuels	Solid fuels	Total	Clean fuels	Solid fuels
Number of participants, *n* (%)	8,563 (100)	2013 (23.50)	6,550 (76.50)	8,563 (100)	3,202 (37.40)	5,361 (62.60)
Age, years (mean ± SD)	58.50 ± 9.06	57.50 ± 9.05	58.70 ± 9.05	58.50 ± 9.06	57.40 ± 8.89	59.10 ± 9.10
Social activity score (mean ± SD)	1.11 ± 1.51	1.44 ± 1.75	1.00 ± 1.41	1.11 ± 1.51	1.35 ± 1.71	0.96 ± 1.35
Sex (%)						
Female	53.60	53.80	53.60	53.60	53.10	54.00
Male	46.40	46.20	46.40	46.40	46.90	46.00
Educational level (%)						
Junior high school or below	90.70	84.90	92.50	90.70	84.60	94.33
Senior high school or above	9.30	15.10	7.50	9.30	15.40	5.67
Socioeconomic status (%)						
Poor	44.70	37.00	47.10	44.70	38.30	48.50
Fair	52.60	59.20	50.60	52.60	57.90	49.40
Good	2.70	3.80	2.30	2.70	3.80	2.10
Residence (%)						
Urban	31.60	56.00	24.20	31.60	51.50	19.80
Rural	68.40	44.00	75.80	68.40	48.50	80.20
Social activity score (%)						
0 (no social activities)	55.10	47.60	57.50	55.10	49.60	58.40
1–2 (infrequent social activities)	21.70	22.20	21.50	21.70	22.10	21.50
≥3 (frequent social activities)	23.20	30.20	21.00	23.20	28.30	20.10
Smoking (%)						
Nonsmoker	61.20	64.50	60.20	61.20	62.80	60.30
Current or former smoker	38.80	35.50	39.80	38.80	37.20	39.70
Drinking (%)						
Nondrinker	61.10	59.70	61.50	61.10	59.90	61.80
Current or former drinker	38.90	40.30	38.50	38.90	40.10	38.20
Marital status (%)						
Married	88.50	89.00	88.30	88.50	89.20	88.00
Divorced	0.60	0.99	0.47	0.60	0.81	0.47
Widowed	10.10	9.74	10.20	10.10	9.78	10.30
Unmarried	0.80	0.27	1.03	0.80	0.21	1.23
Diabetes (%)						
No	93.20	92.40	93.50	93.20	92.20	93.90
Yes	6.80	7.60	6.50	6.80	7.80	6.10
Hypertension (%)						
No	72.90	73.30	72.70	72.90	72.70	73.00
Yes	27.10	26.70	27.30	27.10	27.30	27.00
Stroke (%)						
No	97.30	97.50	97.30	97.33	97.60	97.20
Yes	2.70	2.50	2.70	2.67	2.40	2.80
Cognitive frailty (%)						
No	98.50	99.50	98.20	98.50	99.30	98.0
Yes	1.50	0.50	1.80	1.50	0.70	2.00

All values are presented as proportion (%), or mean (standard deviation). SD, standard deviation.

### Associations between household solid fuel use and cognitive frailty

In the longitudinal analysis, participants who utilized solid fuels for cooking or heating exhibited a higher risk of CF when compared to those employing clean fuels. Table 2 illustrated that the use of solid fuels for cooking significantly increased the risk of CF after adjusting for all covariates (HR: 2.02, 95%CI: 1.25–3.25). A similarly significant association was observed in the use of solid fuels for heating (HR: 2.38, 95%CI: 1.26–4.48). When examining the combined exposure to fuels, individuals reporting the use of solid fuels for both cooking and heating had a significantly higher risk of CF, with HR of 3.17 (95%CI: 1.43–7.05), compared to those reporting the use of clean fuels for both. However, null associations were observed among other combinations of fuel usage.

**Table 2:** Hazard ratios and 95% CI of household solid fuel use for cognitive frailty.

Exposure	Cases	Number of events	Incidence rate per 1,000 person-years (95% CI)	Model 1^a^ HR^d^ (95% CI), *p*-value	Model 2^b^ HR^d^ (95% CI), *P*-value	Model 3^c^ HR^d^ (95% CI), *P*-value
Heating						
Clean fuels	2013	11	1.37 (0.72, 2.53)	**1 (Ref.)**	**1 (Ref.)**	**1 (Ref.)**
Solid fuels	6,550	120	4.63 (3.85, 5.55)	3.28 (1.82, 6.27) <**0.001**	3.10 (1.67, 5.75) <**0.001**	2.38 (1.26, 4.48) **0.007**
Cooking						
Clean fuels	3,202	22	1.72 (1.11, 2.66)	**1 (Ref.)**	**1 (Ref.)**	**1 (Ref.)**
Solid fuels	5,361	109	5.14 (4.24, 6.22)	2.99 (1.89, 4.73) <**0.001**	2.56 (1.62, 4.06) <**0.001**	2.02 (1.25, 3.25) **0.003**
Mixed fuel use						
Both clean fuels	1,589	7	1.10 (0.48, 2.38)	**1 (Ref.)**	**1 (Ref.)**	**1 (Ref.)**
Clean fuel use for cooking and solid fuel use for heating	1,598	15	2.34 (1.36, 3.95)	2.12 (0.45, 1.64) 0.10	2.15 (0.87, 5.29) 0.09	1.67 (0.66, 4.23) 0.27
Solid fuel use for cooking and clean fuel use for heating	424	4	2.38 (0.76, 6.52)	2.15 (0.63, 7.37) 0.22	1.79 (0.52, 6.18) 0.35	1.39 (0.37, 5.20) 0.62
Solid fuel use for both cooking and heating	4,937	105	5.38 (4.42, 6.54)	4.89 (2.27, 10.52) **<0.001**	4.20 (1.95, 9.03) **<0.001**	3.17 (1.43, 7.05) **0.004**

Model 1^a^: no covariates were adjusted; Model 2^b^: adjusted for age and sex; Model 3^c^: age, sex, education level, marital status, smoking, drinking status, residence, self-reported socioeconomic status, social activities, hypertension, diabetes, and stroke; HR^d^: effect value; Bolded *p*-values indicate statistical significance. HR, hazard ratio; 95% CI, 95% confidence interval.

### Associations between fuel use switching and cognitive frailty

Table 3 illustrated that 1,045 individuals transitioned from solid to clean fuels for cooking and 393 for heating, with their CF incidence rates (per 1,000 person-years) recorded at 2.24 and 1.70, respectively. When compared with counterparts who continued using solid fuels for cooking, those who made the switch to clean fuels demonstrated a significant reduction in CF risk (HR: 0.38, 95%CI: 0.16–0.88). A similar association was reported among individuals switching from solid to clean heating fuels in Models 1 and 2. Conversely, 2,174 individuals transformed from clean to solid cooking fuels, and 3,152 made a similar switch for heating, with their CF incidence rates (per 1,000 person-years) estimated to be 6.51 and 5.44, respectively. Compared to those who consistently used clean fuels for cooking, the group that switched from clean to solid fuels had an increased risk of developing CF (HR: 2.45, 95% CI: 1.16–5.19).

**Table 3:** Incidence rates and adjusted hazard ratios for cognitive frailty in association with switching fuel types.

Exposure	Cases	Number of events	Incidence rate per 1,000 person-years (95% CI)	Model 1^a^ HR^d^ (95% CI), *p*-value	Model 2^b^ HR^d^ (95% CI), *p*-value	Model 3^c^ HR^d^ (95% CI), *P*-value
Cooking						
Solid fuel use	1879	36	6.47 (4.60–9.15)	**1 (Ref.)**	**1 (Ref.)**	**1 (Ref.)**
Solid to clean fuel use	1,045	7	2.24 (0.98–4.84)	0.34 (0.15, 0.77) **0.01**	0.32 (0.14, 0.72) **0.006**	0.38 (0.16, 0.88) **0.02**
Clean fuel use	1720	9	1.75 (0.85, 3.44)	**1 (Ref.)**	**1 (Ref.)**	**1 (Ref.)**
Clean to solid fuel use	2,174	42	6.51 (4.75, 8.87)	3.72 (1.81, 7.66) **<0.001**	3.35 (1.63, 6.90) **0.001**	2.45 (1.16, 5.19) **0.01**
Heating						
Solid fuel use	338	9	8.99 (4.39, 17.65)	**1 (Ref.)**	**1 (Ref.)**	**1 (Ref.)**
Solid to clean fuel use	393	2	1.70 (0.29, 6.83)	0.18 (0.04, 0.87) **0.03**	0.20 (0.04, 0.96) **0.04**	0.25 (0.05, 1.26) 0.09
Clean fuel use	428	1	0.77 (0.04, 5.04)	**1 (Ref.)**	**1 (Ref.)**	**1 (Ref.)**
Clean to solid fuel use	3,152	51	5.44 (4.10, 7.21)	7.00 (0.96, 50.67) 0.053	6.12 (0.84, 44.33) 0.07	4.46 (0.60, 32.82) 0.14

Model 1^a^: no covariates were adjusted; Model 2^b^: adjusted for age and sex; Model 3^c^: age, sex, education level, social activities, marital status, smoking, drinking status, residence, self-reported socioeconomic status, hypertension, diabetes, and stroke; HR^d^: effect value; Bolded P-values indicate statistical significance. HR, hazard ratio; 95% CI, 95% confidence interval.

### Sensitivity analyses

We conducted a series of sensitivity tests to affirm the robustness of our results. Firstly, when central heating was additionally included in the original fuel use, the results remained consistent with previous findings (Supplementary Table S1). Moreover, when analyzing solid fuels (coal and crop residues/wood burning) separately, the results remained stable (Supplementary Tables S2, S3). A similar trend was observed for the use of fuels for heating purposes (HR: 0.21, 95% CI: 0.05–0.86). Finally, stratified analysis revealed variations in how influenced the results (Supplementary Tables S4–S9): despite overall consistency in outcomes when analyzed by stratification, a significant association was found within the group aged 65 and below, but not in those over 65. Similarly, a significant association was observed in groups with lower socioeconomic status, while no such association existed within groups of higher socioeconomic status. Notably, the use of solid fuels was significantly associated with an increased risk of developing CF in both smokers and non-smokers. Concurrently, no statistically significant interactions were observed among participants stratified by age, sex, residence, socioeconomic status, smoking, and drinking (all *P* for interaction >0.05).

## Discussion

Our study demonstrates a significant association between the use of indoor solid fuels and an increased risk of CF compared to clean fuels. Both cooking and heating with solid fuels are linked to a higher risk of CF, with the risk being even greater when solid fuels are used for both activities. This suggests that combined exposure to solid fuels for cooking and heating may result in a higher cumulative exposure to harmful pollutants, thereby increasing the risk of CF.

Our study has found a significant link between solid fuel usage and CF among middle-aged and older adult groups, consistent with earlier research findings. Specifically, by examining data from individuals aged 50 and above, three national cohort studies utilized time-dependent Cox regression models and mediation effect analyses (18). These studies provided robust evidence that the use of solid fuels for cooking markedly elevates the risk of CI. Moreover, an extensive analysis of 7,824 individuals in middle age and older from China, evaluating cognitive abilities via standardized surveys, showed that the use of solid fuels for cooking and heating markedly elevated the risk of CI (34). Cao and colleagues conducted a longitudinal study with 4,946 older adult individuals and discovered a positive correlation between the use of solid cooking fuels and the incidence of PF, in line with prior research findings (35). Furthermore, an analysis leveraging prospective data from CHARLS, which included 4,685 participants initially non-frail, identified a positive link between the use of solid fuels and a heightened risk of frailty (36). Additionally, one research conducted on 4,535 older adult individuals from 23 provinces substantiated the link between solid fuel use and a significant increase in the frailty index (37). Taken together, these results substantially reinforce the credibility and strength of our findings, confirming the harmful effects of solid fuel usage on the health of middle-aged and older adults.

To our knowledge, this study represents the inaugural cohort analysis exploring the link between domestic solid fuel consumption and CF. However, the exact mechanism of this association is unclear. CF is defined as a coexistence of CI and PF (1). Based on this definition, the potential mechanisms of impact may encompass: Regarding the impact of solid fuels on cognitive function, it was initially found that indoor air pollution, especially resulting from solid fuel combustion, is known to elevate the risk of respiratory and cardiovascular conditions (38, 39), both of which are known risk factors for CI (40). Secondly, pollutants from solid fuel burning can disrupt the regulatory roles of brain capillaries and initiate pro-inflammatory reactions, causing pathological alterations in the central nervous system and additional CI (41). Additionally, toxic pollutants released by the combustion of solid fuels, such as particulate matter, carbon monoxide, and sulfur dioxide (42), might impact protein aggregation through oxidative stress mechanisms, interfering with early biomarkers of neurodegenerative diseases, such as soluble Aβ and *α*-synuclein, thereby causing cognitive damage (41, 43, 44). Finally, long-term use of solid fuels is associated with reduced insulin levels (45), which could lead to inflammation, abnormal energy metabolism, altered vascular function, and reduced synaptic activity, ultimately resulting in a decline in cognitive function (46). Concerning the effect of solid fuels on PF, studies have shown a strong positive link between solid fuel usage and the incidence of PF in older populations (47). To begin with, PM2.5 particulates generated from solid fuels are closely linked with heightened systemic inflammation, increased platelet activation, and a decrease in erythrocyte antioxidant enzyme activity (48). These factors are key in the progression of PF. Additionally, HAP resulting from solid fuel combustion can hasten the aging process of cells, organs, and systemic functions, ultimately contributing to PF (49). Moreover, prolonged use of solid fuels is found to be directly associated with a heightened risk of arthritis in individuals aged 45 and above (29), a condition frequently identified as an indicator of PF (50). Lastly, long-term exposure to solid fuels correlates with decreased insulin levels, potentially leading to impaired skeletal muscle metabolism and contraction (40) and thereby precipitating the onset of PF (51).

In our stratified analysis, we observed a consistent trend: among smokers, the risk of CF increases regardless of the type of solid fuel used. This finding aligns with previous research, illustrating a broader pattern of risk. For example, population surveys on HAP in Northern China have shown that individuals who smoke and use solid fuels for cooking or heating display lower cognitive abilities. This suggested a compounded effect of smoking and solid fuel use on cognitive health (52). Likewise, analysis of data from rural communities showed a notable rise in CI risk among individuals who smoke and utilize solid fuels, further highlighting the connection between these risk elements and cognitive deterioration (35). A study from India supports this conclusion, showing that smokers generally have diminished cognitive abilities, highlighting the intrinsic risk smoking poses to cognitive well-being (53). The underlying mechanisms for these observations are complex. Harmful chemicals in tobacco smoke may induce neurotoxic effects, leading directly to cognitive deterioration (54). Exposure to smoke can also inhibit neurogenesis and promote glial cell proliferation in the dentate gyrus, which might work in synergy with the decrease in dopaminergic neurons seen with solid fuel use (55). Moreover, tobacco smoke and the free radicals from solid fuel combustion can trigger inflammatory responses, damaging the central nervous system and increasing CI risk (56). Importantly, research has shown that smoking leads to muscle atrophy and reduced resistance to muscle fatigue, potentially impairing physical function (57). Epidemiological evidence indicates that HAP from tobacco and solid fuels can cause various organ damages, accelerate cellular aging, trigger skeletal muscle dysfunction, and heighten the risk of chronic diseases, muscle atrophy, and sarcopenia (58, 59). Furthermore, inhaling smoke from these sources may elevate C-reactive protein levels, correlating with decreased physical function and exacerbating PF (60). Stratified analyses further observe that individuals aged between 45 and 65 are particularly sensitive to the effects of solid fuel use despite the lack of direct evidence. A possible mechanism, according to World Health Organization surveys, is that individuals aged 65 and older often see a decrease in daily activities and an uptick in sedentary behavior. This leads to a lifestyle that becomes more monotonous and potentially less engaging (61). Such a change may result in less frequent use of solid fuels, thereby reducing the risk of cognitive decline associated with their use.

This research possesses several significant strengths. Secondly, it includes a nationally representative cohort with long-term follow-up, enhancing the relevance of our findings for the middle-aged and older demographics in China. What’s more, we employed different Cox regression models and referenced past literature, incorporating relevant confounding factors to minimize the potential impact of covariates on our findings. Subsequently, our study primarily centered on CF, an outcome that encompasses cognitive function while also considering the impact of physical health on cognition. This in-depth analysis not only aligns closely with clinical practice but also enhances the thoroughness and depth of our findings. Lastly, by performing multiple sensitivity analyses, we have further validated the consistency and trustworthiness of our outcomes.

However, several limitations warrant attention. Firstly, the dependence on self-reported indoor fuel usage may introduce recall bias, potentially leading to misclassification errors. Concurrently, employing self-reports of cooking and heating with solid fuels as indicators for HAP exposure may not precisely capture the actual exposure levels, given that variations can arise from factors like the effectiveness of ventilation, weather conditions, and humidity levels. Moreover, considering CF encompasses both PF and CI, our analysis did not explore the specific relationships between household solid fuel use and these individual components. Furthermore, while adjustments were made to covariates as comprehensively as possible, based on existing literature, it remains challenging to account for all potential confounders. For example, household ventilation, an influential covariate, was not assessed in the CHARLS. Significantly, the pronounced disparities in the prevalence of conditions such as hypertension, diabetes, and stroke between diagnosed and undiagnosed individuals precluded further stratified analyses for these conditions, aiming to enhance the rigor of our findings. Lastly, the limited sample size led to wide confidence intervals in some stratified analyses. Thus, our findings should be considered with caution. Future research in a large population is essential to validate our initial findings.

## Conclusion

This is the first longitudinal study among the Chinese population to explore the association between solid fuel use and CF. Our cohort research reveals a significant link between HAP from solid fuel use and a higher incidence of CF in middle-aged and older adult groups in China. CF is considered a reversible condition. For individuals, our study underscores the importance of personal protection during solid fuel use and early intervention to prevent CF in older adults. At a societal level, the study highlights the harmful effects of solid fuel use and offers a novel perspective on population health, advocating for a transition from solid fuels to clean fuels. Future research should involve larger, population-based cohorts with longer follow-up periods to better characterize fuel composition and individual exposure. Additionally, toxicological studies are needed to provide experimental evidence supporting these findings.

## Data Availability

Publicly available datasets were analyzed in this study. This data can be found here: The data that support the findings of this study are available from the website of China Health and Retirement Longitudinal Study at: http://charls.pku.edu.cn/en.

## Footnotes

^1^ http://charls.pku.edu.cn/en

## References

[1] KelaiditiECesariMCanevelliMAbellan Van KanGOussetPJGillette-GuyonnetS. Cognitive frailty: rational and definition from an (I.A.N.A./I.A.G.G.) international consensus group. J Nutr Health Aging. (2013) 17:726–34. doi: 10.1007/s12603-013-0367-2, PMID: 24154642

[2] Robertson DA Savva GM Kenny RA. Frailty and cognitive impairment—a review of the evidence and causal mechanisms. Ageing Res Rev. (2013) 12:840–51. doi: 10.1016/j.arr.2013.06.004 23831959

[3] Shimada H Makizako H Doi T Yoshida D Tsutsumimoto K Anan Y. Combined prevalence of frailty and mild cognitive impairment in a population of elderly Japanese people. J Am Med Dir Assoc. (2013) 14:518–24. doi: 10.1016/j.jamda.2013.03.010 23669054

[4] Arai H Satake S Kozaki K. Cognitive frailty in geriatrics. Clin Geriatr Med. (2018) 34:667–75. doi: 10.1016/j.cger.2018.06.011 30336994

[5] Chen C Park JY Wu CK Xue QL Agogo G Han L. Cognitive frailty in relation to adverse health outcomes independent of multimorbidity: results from the China health and retirement longitudinal study. Aging. (2020) 12:23129–45. doi: 10.18632/aging.104078 33221750 PMC7746379

[6] Sugimoto T Sakurai T Ono R Kimura A Saji N Niida S. Epidemiological and clinical significance of cognitive frailty: a mini review. Ageing Res Rev. (2018) 44:1–7. doi: 10.1016/j.arr.2018.03.002 29544875

[7] Shimada H Doi T Lee S Makizako H Chen L-K Arai H. Cognitive frailty predicts incident dementia among community-dwelling older people. J Clin Med. (2018) 7:250. doi: 10.3390/jcm7090250 30200236 PMC6162851

[8] O’caoimh R Sezgin D Odonovan MR Molloy DW Clegg A Rockwood K. Prevalence of frailty in 62 countries across the world: a systematic review and meta-analysis of population-level studies. Age Ageing. (2020) 50:96–104. doi: 10.1093/ageing/afaa219 33068107

[9] Verghese J Lipton RB Katz MJ Hall CB Derby CA Kuslansky G. Leisure activities and the risk of dementia in the elderly. N Engl J Med. (2003) 348:2508–16. doi: 10.1056/NEJMoa022252 12815136

[10] Du M Tao LY Liu M Liu J. Trajectories of health conditions and their associations with the risk of cognitive impairment among older adults: insights from a national prospective cohort study. BMC Med. (2024) 22:20. doi: 10.1186/s12916-024-03245-x 38195549 PMC10777570

[11] Wang ZQ Dong BR. Screening for cognitive impairment in geriatrics. Clin Geriatr Med. (2018) 34:515–36. doi: 10.1016/j.cger.2018.06.004 30336986

[12] Yuan MQ Xu CH Fang Y. The transitions and predictors of cognitive frailty with multi-state Markov model: a cohort study. BMC Geriatr. (2022) 22:550. doi: 10.1186/s12877-022-03220-2 35778705 PMC9248089

[13] Feng L Zin Nyunt MS Gao Q Feng L Yap KB Ng T-P. Cognitive frailty and adverse health outcomes: findings from the Singapore longitudinal ageing studies (SLAS). J Am Med Dir Assoc. (2017) 18:252–8. doi: 10.1016/j.jamda.2016.09.015 27838339

[14] Liu YH Zeng SL Huang C Wang C Zhu JJ Peng JH. Indoor solid fuel use and non-neoplastic digestive system diseases: a population-based cohort study among Chinese middle-aged and older population. Int J Public Health. (2022) 67:1605419. doi: 10.3389/ijph.2022.1605419 36618433 PMC9810631

[15] Adekoya A Tyagi SK Duru CN Satia I Paudyal V Kurmi OP. Effects of household air pollution (HAP) on cardiovascular diseases in low-and middle-income countries (LMICs): a systematic review and meta-analysis. Int J Environ Res Public Health. (2022) 19:9298. doi: 10.3390/ijerph19159298 35954653 PMC9368384

[16] Lin BQ Wei K. Does use of solid cooking fuels increase family medical expenses in China? Int J Environ Res Public Health. (2022) 19:1649. doi: 10.3390/ijerph19031649 35162671 PMC8835481

[17] Liu Y Chen X Yan ZJ. Depression in the house: the effects of household air pollution from solid fuel use among the middle-aged and older population in China. Sci Total Environ. (2020) 703:134706. doi: 10.1016/j.scitotenv.2019.134706 31731156 PMC9420076

[18] Hahad O Lelieveld J Birklein F Lieb K Daiber A Münzel T. Ambient air pollution increases the risk of cerebrovascular and neuropsychiatric disorders through induction of inflammation and oxidative stress. Int J Mol Sci. (2020) 21:4306. doi: 10.3390/ijms21124306 32560306 PMC7352229

[19] Zhao Y Hu Y Smith JP Strauss J Yang G. Cohort profile: the China health and retirement longitudinal study (CHARLS). Int J Epidemiol. (2012) 43:61–8. doi: 10.1093/ije/dys203 23243115 PMC3937970

[20] Chen W Wang XY Chen J You C Ma L Zhang W. Household air pollution, adherence to a healthy lifestyle, and risk of cardiometabolic multimorbidity: results from the China health and retirement longitudinal study. Sci Total Environ. (2023) 855:158896. doi: 10.1016/j.scitotenv.2022.158896 36150596

[21] Xu TT Ye XY Lu XL Lan GH Xie MY Huang ZL. Association between solid cooking fuel and cognitive decline: three nationwide cohort studies in middle-aged and older population. Environ Int. (2023) 173:107803. doi: 10.1016/j.envint.2023.107803 36805161

[22] Liu Y Ning N Sun T Guan HC Liu ZY Yang WS. Association between solid fuel use and nonfatal cardiovascular disease among middle-aged and older adults: findings from the China health and retirement longitudinal study (CHARLS). Sci Total Environ. (2023) 856:159035. doi: 10.1016/j.scitotenv.2022.159035 36191716

[23] Fried LP Tangen CM Walston J Newman AB Hirsch C Gottdiener J. Frailty in older adults: evidence for a phenotype. J Gerontol A Biol Sci Med Sci. (2001) 56:M146–57. doi: 10.1093/gerona/56.3.M146 11253156

[24] Wu CK Smit E Xue QL Odden MC. Prevalence and correlates of frailty among community-dwelling Chinese older adults: the China health and retirement longitudinal study. J Gerontol A Biol Sci Med Sci. (2017) 73:102–8. doi: 10.1093/gerona/glx098 28525586 PMC5861883

[25] Zhu XH Ding LL Zhang XN Xiong ZF. Association of cognitive frailty and abdominal obesity with cardiometabolic multimorbidity among middle-aged and older adults: a longitudinal study. J Affect Disord. (2023) 340:523–8. doi: 10.1016/j.jad.2023.08.067 37595895

[26] Sha S Pan Y Xu YB Chen L. Associations between loneliness and frailty among older adults: evidence from the China health and retirement longitudinal study. BMC Geriatr. (2022) 22:537. doi: 10.1186/s12877-022-03044-0 35773656 PMC9247968

[27] Li CX Jin SY Cao XQ Han L Sun N Allore H. Catastrophic health expenditure among Chinese adults living alone with cognitive impairment: findings from the CHARLS. BMC Geriatr. (2022) 22:640. doi: 10.1186/s12877-022-03341-8 35922775 PMC9351200

[28] Deng Y Gao Q Yang TY Wu B Liu Y Liu RX. Indoor solid fuel use and incident arthritis among middle-aged and older adults in rural China: a nationwide population-based cohort study. Sci Total Environ. (2021) 772:145395. doi: 10.1016/j.scitotenv.2021.145395 33578144

[29] Luo JH Zhang TM Yang LL Cai YY Yang Y. Association between relative muscle strength and hypertension in middle-aged and older Chinese adults. BMC Public Health. (2023) 23:2087. doi: 10.1186/s12889-023-17007-6 37880652 PMC10598916

[30] Liu Y Shao JN Liu QT Zhou WH Huang R Zhou J. Association between household fuel combustion and diabetes among middle-aged and older adults in China: a cohort study. Ecotoxicol Environ Saf. (2023) 258:114974. doi: 10.1016/j.ecoenv.2023.114974 37150109

[31] Cox DR. Regression models and life-tables. J R Stat Soc B. (1972) 34:187–202. doi: 10.1111/j.2517-6161.1972.tb00899.x

[32] Schoenfeld D. Partial residuals for the proportional hazards regression model. Biometrika. (1982) 69:239–41. doi: 10.1093/biomet/69.1.239

[33] Zhao XY Ruan ZL Tian Y Du W Fan LJ. Estimating the joint effect of household solid fuel use and social isolation on depression among middle-aged and older adults in China. Sci Total Environ. (2023) 901:166411. doi: 10.1016/j.scitotenv.2023.166411 37611698

[34] Chen HY Chen L Hao G. Sex difference in the association between solid fuel use and cognitive function in rural China. Environ Res. (2021) 195:110820. doi: 10.1016/j.envres.2021.110820 33539833

[35] Cao LM Zhao ZY Ji C Xia Y. Association between solid fuel use and cognitive impairment: a cross-sectional and follow-up study in a middle-aged and older Chinese population. Environ Int. (2021) 146:106251. doi: 10.1016/j.envint.2020.106251 33248346

[36] Jin XY Wang YL Wu YD Liang YF Li YX Sun XN. The increased medical burden associated with frailty is partly attributable to household solid fuel: a nationwide prospective study of middle-aged and older people in China. Sci Total Environ. (2023) 858:159829. doi: 10.1016/j.scitotenv.2022.159829 36374752

[37] Chen HY Xu XP Jia CC Gu H Zhang L Yi Y. Household polluting fuel use and frailty among older adults in rural China: the moderating role of healthy lifestyle behaviors. Healthcare. (2023) 11:1747. doi: 10.3390/healthcare11121747 37372865 PMC10298731

[38] Huang SB Guo CM Qie RR Han MH Wu XY Zhang YY. Solid fuel use and cardiovascular events: a systematic review and meta-analysis of observational studies. Indoor Air. (2021) 31:1722–32. doi: 10.1111/ina.12867 34110043

[39] Chan KH Kurmi OP Bennett DA Yang L Chen Y Tan Y. Solid fuel use and risks of respiratory diseases. A cohort study of 280,000 Chinese never-smokers. Am J Respir Crit Care Med. (2019) 199:352–61. doi: 10.1164/rccm.201803-0432OC 30235936 PMC6363974

[40] Kalaria RN Akinyemi R Ihara M. Stroke injury, cognitive impairment and vascular dementia. BBA Mol Basis Dis. (2016) 1862:915–25. doi: 10.1016/j.bbadis.2016.01.015 PMC4827373 26806700

[41] Block ML Calderón-Garcidueñas L. Air pollution: mechanisms of neuroinflammation and CNS disease. Trends Neurosci. (2009) 32:506–16. doi: 10.1016/j.tins.2009.05.009 19716187 PMC2743793

[42] Shi TT Liu YQ Zhang LB Hao L Gao ZQ. Burning in agricultural landscapes: an emerging natural and human issue in China. Landsc Ecol. (2014) 29:1785–98. doi: 10.1007/s10980-014-0060-9

[43] Altuğ H Fuks KB Hüls A Mayer A-K Tham R Krutmann J. Air pollution is associated with depressive symptoms in elderly women with cognitive impairment. Environ Int. (2020) 136:105448. doi: 10.1016/j.envint.2019.105448 31931346

[44] Hajipour S Farbood Y Gharib-Naseri MK Goudarzi G Rashno M Maleki H. Exposure to ambient dusty particulate matter impairs spatial memory and hippocampal LTP by increasing brain inflammation and oxidative stress in rats. Life Sci. (2020) 242:117210. doi: 10.1016/j.lfs.2019.117210 31874166

[45] Kang N Song XQ Zhang CY Li RY Yuchi YH Liao W. Association of household air pollution with glucose homeostasis markers in Chinese rural women: effect modification of socioeconomic status. Ecotoxicol Environ Saf. (2022) 248:114283. doi: 10.1016/j.ecoenv.2022.114283 36371884

[46] Kellar D Craft S. Brain insulin resistance in Alzheimer's disease and related disorders: mechanisms and therapeutic approaches. Lancet Neurol. (2020) 19:758–66. doi: 10.1016/S1474-4422(20)30231-3 32730766 PMC9661919

[47] Cao LM Zhai DK Kuang MJ Xia Y. Indoor air pollution and frailty: a cross-sectional and follow-up study among older Chinese adults. Environ Res. (2022) 204:112006. doi: 10.1016/j.envres.2021.112006 34499891

[48] Delfino RJ Staimer N Tjoa T Gillen DL Polidori A Arhami M. Air pollution exposures and circulating biomarkers of effect in a susceptible population: clues to potential causal component mixtures and mechanisms. Environ Health Perspect. (2009) 117:1232–8. doi: 10.1289/ehp.0800194 19672402 PMC2721866

[49] Fedarko NS. The biology of aging and frailty. Clin Geriatr Med. (2011) 27:27–37. doi: 10.1016/j.cger.2010.08.006 21093720 PMC3052959

[50] Taylor JA Greenhaff PL Bartlett DB Jackson TA Duggal NA Lord JM. Multisystem physiological perspective of human frailty and its modulation by physical activity. Physiol Rev. (2023) 103:1137–91. doi: 10.1152/physrev.00037.2021 36239451 PMC9886361

[51] Clegg A Hassan-Smith Z. Frailty and the endocrine system. Lancet Diabetes Endocrinol. (2018) 6:743–52. doi: 10.1016/S2213-8587(18)30110-4 30017798

[52] Tseng T-WJ Carter E Yan L Chan Q Elliott P Ezzati M. Household air pollution from solid fuel use as a dose-dependent risk factor for cognitive impairment in northern China. Sci Rep. (2022) 12:6187. doi: 10.1038/s41598-022-10074-6 35418188 PMC9008006

[53] Mandi R Bansod DW Goyal AK. Exploring the association of lifestyle behaviors and healthy ageing among the older adults in India: evidence from LASI survey. BMC Geriatr. (2023) 23:675. doi: 10.1186/s12877-023-04367-2 37853323 PMC10585826

[54] SBA G Choi S Krishnan J. Cigarette smoke and related risk factors in neurological disorders: an update. Biomed Pharmacother. (2017) 85:79–86. doi: 10.1016/j.biopha.2016.11.118 27930990

[55] Bruijnzeel AW Bauzo RM Munikoti V Rodrick GB Yamada H Fornal CA. Tobacco smoke diminishes neurogenesis and promotes gliogenesis in the dentate gyrus of adolescent rats. Brain Res. (2011) 1413:32–42. doi: 10.1016/j.brainres.2011.07.041 21840504

[56] Kamal A Cincinelli A Martellini T Malik RN. A review of PAH exposure from the combustion of biomass fuel and their less surveyed effect on the blood parameters. Environ Sci Pollut Res. (2015) 22:4076–98. doi: 10.1007/s11356-014-3748-0 25410307

[57] Darabseh MZ Maden-Wilkinson TM Welbourne G Wüst RCI Ahmed N Aushah H. Fourteen days of smoking cessation improves muscle fatigue resistance and reverses markers of systemic inflammation. Sci Rep. (2021) 11:12286. doi: 10.1038/s41598-021-91510-x 34112815 PMC8192509

[58] Burke KE. Mechanisms of aging and development—a new understanding of environmental damage to the skin and prevention with topical antioxidants. Mech Ageing Dev. (2018) 172:123–30. doi: 10.1016/j.mad.2017.12.003 29287765

[59] Zhang B Huang L Zhu X Ran L Zhao H Zhu Z. Impact of household solid fuel use on sarcopenia in China: a nationwide analysis. Sci Total Environ. (2023) 877:162814. doi: 10.1016/j.scitotenv.2023.162814 36933714

[60] Liu Q Gu X Deng F Mu L Baccarelli AA Guo X. Ambient particulate air pollution and circulating C-reactive protein level: a systematic review and meta-analysis. Int J Hyg Environ Health. (2019) 222:756–64. doi: 10.1016/j.ijheh.2019.05.005 31103472

[61] World Health Organization. (2023). https://www.who.int/europe/news-room/11-10-2023-by-2024--the-65-and-over-age-group-will-outnumber-the-youth-group--new-who-report-on-healthy-ageing (Accessed December 25, 2024).

