# Supplementary material for: Association between household solid fuel use and cognitive frailty in a middle-aged and older Chinese population
Source: Front Public Health. 2025 Mar 26;13:1444421. doi: 10.3389/fpubh.2025.1444421 (PMC11979103; doi:10.3389/fpubh.2025.1444421)
Supplement: Supplementary file 1 [file Table_1.DOCX]

**Table S1. Association Between Indoor Solid Fuel Use and Cognitive Frailty after the Inclusion of Central Heating.**

| **Exposure** | **Cases** | **Number of events** | **Incidence rate per 1000 person-years (95% CI)** | **Model 1^a^**  **HR^d^ (95% CI), *P*-value** | **Model 2^b^**  **HR^d^ (95% CI), *P*-value** | **Model 3^c^**  **HR^d^ (95% CI), *P*-value** |
| --- | --- | --- | --- | --- | --- | --- |
| **Heating** |  |  |  |  |  |  |
| Clean fuels | 1991 | 11 | 1.39 (0.73, 2.56) | **1 (Ref.)** | **1 (Ref.)** | **1 (Ref.)** |
| Solid fuels | 7328 | 117 | 4.03 (3.35, 4.84) | 2.91 (1.57, 5.40) **<0.001** | 2.66 (1.43, 4.95) **0.001** | 2.16 (1.15, 4.05) **0.015** |
| **Cooking** |  |  |  |  |  |  |
| Clean fuels | 3924 | 26 | 1.66 (1.11, 2.47) | **1 (Ref.)** | **1 (Ref.)** | **1 (Ref.)** |
| Solid fuels | 5395 | 102 | 4.78 (3.92, 5.82) | 2.88 (1.87, 4.43) **<0.001** | 2.58 (1.68, 3.98) **<0.001** | 1.90 (1.20, 3.00) **0.005** |
|  |  |  |  |  |  |  |
| **Mixed fuel use** |  |  |  |  |  |  |
| Both clean fuels | 1571 | 7 | 1.12 (0.48, 2.41) | **1 (Ref.)** | **1 (Ref.)** | **1 (Ref.)** |
| Clean fuel use for cooking and solid fuel use for heating | 2353 | 19 | 2.03 (1.26, 3.23) | 1.81 (0.76, 4.32) 0.17 | 1.70 (0.71, 4.06) 0.22 | 1.73 (0.72, 4.14) 0.22 |
| Solid fuel use for cooking and clean fuel use for heating | 420 | 4 | 2.40 (0.77, 6.59) | 2.15 (0.63, 7.36) 0.22 | 1.77 (0.51, 6.10) 0.36 | 1.36 (0.36, 5.06) 0.64 |
| Solid fuel use for both cooking and heating | 4975 | 98 | 4.98 (4.06, 6.09) | 4.48 (2.08, 9.64) **<0.001** | 3.86 (1.79, 8.33) **<0.001** | 3.02 (1.35, 6.74) **0.006** |

**Notes**: **Model 1^a^**: no covariates were adjusted; **Model 2^b^:** adjusted for age and sex; **Model 3^c^:** age, sex, education level, marital status, smoking, drinking status, residence, self-reported socioeconomic status, hypertension, diabetes, and stroke; **HR^d^**: effect value;

Bolded *P*-values indicate statistical significance. **Abbreviations: HR:** hazard ratio**; 95% CI:** 95% confidence interval

**Table S2. Analysis of the Independent Impacts of Cooking Solid Fuel Use on Cognitive Frailty.**

| **Cook fuel** | **Coal use** |  |  | **Crop residue/Wood burning use** | |
| --- | --- | --- | --- | --- | --- |
|  | **HR (95% CI)** | ***P*-value** |  | **HR (95% CI)** | ***P*-value** |
| **Household fuel types** |  |  |  |  |  |
| Heating |  |  |  |  |  |
| Clean fuels | **1 (Ref.)** |  |  | **1 (Ref.)** |  |
| Solid fuels | 2.13 (0.92, 4.91) | 0.07 |  | 2.18 (1.14, 4.16) | **0.017** |
| Cooking |  |  |  |  |  |
| Clean fuels | **1 (Ref.)** |  |  | **1 (Ref.)** |  |
| Solid fuels | 1.76 (0.93, 3.32) | 0.08 |  | 2.03 (1.24, 3.33) | **0.004** |

**Note**: Model adjusted for age, sex, education level, marital status, smoking, drinking status, residence, self-reported socioeconomic status, hypertension, diabetes, and stroke; Bolded *P*-values indicate statistical significance.

**Abbreviations: HR:** hazard ratio**; 95% CI:** 95% confidence interval

**Table S3. Analysis of the Independent Impacts of Heating Solid Fuel Use on Cognitive Frailty.**

| **Heat fuel** | **Coal use** |  |  | **Crop residue/Wood burning use** | |
| --- | --- | --- | --- | --- | --- |
|  | **HR (95% CI)** | ***P*-value** |  | **HR (95% CI)** | ***P*-value** |
| **Household fuel types** |  |  |  |  |  |
| Heating |  |  |  |  |  |
| Clean fuels | **1 (Ref.)** |  |  | **1 (Ref.)** |  |
| Solid fuels | 2.42 (1.24, 4.71) | **0.009** |  | 2.26 (1.14, 4.48) | **0.02** |
| Cooking |  |  |  |  |  |
| Clean fuels | **1 (Ref.)** |  |  | **1 (Ref.)** |  |
| Solid fuels | 2.13 (1.17, 3.86) | **0.01** |  | 1.98 (1.05, 3.71) | **0.03** |

**Note**: Model adjusted for age, sex, education level, marital status, smoking, drinking status, residence, self-reported socioeconomic status, hypertension, diabetes, and stroke; Bolded *P*-values indicate statistical significance.

**Abbreviations: HR:** hazard ratio**; 95% CI:** 95% confidence interval

**Table S4: Hazard Ratios and 95% Confidence Intervals for Cognitive Frailty Related to Household Solid Fuel Use Stratified by Age Categories.**

|  | **Age <= 65 y (n = 6730)** | |  | **Age > 65 y (n = 1833)** | |
| --- | --- | --- | --- | --- | --- |
|  | **HR (95% CI)** | ***P*-value** |  | **HR (95% CI)** | ***P*-value *P* for interaction** |
| **Household fuel types** |  |  |  |  |  |
| Heating |  |  |  |  | 0.88 |
| Clean fuels | **1 (Ref.)** |  |  | **1 (Ref.)** |  |
| Solid fuels | 5.91 (1.41, 24.71) | **0.014** |  | 1.59 (0.78, 3.27) | 0.19 |
| Cooking |  |  |  |  | 0.24 |
| Clean fuels | **1 (Ref.)** |  |  | **1 (Ref.)** |  |
| Solid fuels | 2.57 (1.17, 5.61) | **0.02** |  | 2.56 (0.97, 3.23) | 0.06 |

**Note**: Model adjusted for sex, education level, marital status, smoking, drinking status, residence, self-reported socioeconomic status, hypertension, diabetes, and stroke; Bolded *P*-values indicate statistical significance.

**Abbreviations: HR:** hazard ratio**; 95% CI:** 95% confidence interval

**Table S5. Hazard Ratios and 95% Confidence Intervals for Cognitive Frailty Related to Household Solid Fuel Use Stratified by Sex Categories.**

|  | **Men (n = 3969)** | |  | **Women (n = 4594)** | |
| --- | --- | --- | --- | --- | --- |
|  | **HR (95% CI)** | ***P*-value** |  | **HR (95% CI)** | ***P*-value *P* for interaction** |
| **Household fuel types** |  |  |  |  |  |
| Heating |  |  |  |  | 0.86 |
| Clean fuels | **1 (Ref.)** |  |  | **1 (Ref.)** |  |
| Solid fuels | 2.95 (1.16, 7.50) | **0.02** |  | 2.37 (1.01, 5.58) | **0.04** |
| Cooking |  |  |  |  | 0.74 |
| Clean fuels | **1 (Ref.)** |  |  | **1 (Ref.)** |  |
| Solid fuels | 2.77 (1.37, 5.58) | **0.004** |  | 2.05 (1.07, 3.90) | **0.03** |

**Note**: Model adjusted for age, education level, marital status, smoking, drinking status, residence, self-reported socioeconomic status, hypertension, diabetes, and stroke; Bolded *P*-values indicate statistical significance.

**Abbreviations: HR:** hazard ratio**; 95% CI:** 95% confidence interval

**Table S6. Hazard Ratios and 95% Confidence Intervals for Cognitive Frailty Related to Household Solid Fuel Use Stratified by Residence Categories.**

|  | **Urban (n = 5853)** | |  | **Rural (n = 2710)** | |
| --- | --- | --- | --- | --- | --- |
|  | **HR (95% CI)** | ***P*-value** |  | **HR (95% CI)** | ***P*-value *P* for interaction** |
| **Household fuel types** |  |  |  |  |  |
| Heating |  |  |  |  | 0.73 |
| Clean fuels | **1 (Ref.)** |  |  | **1 (Ref.)** |  |
| Solid fuels | 2.68 (0.89, 8.06) | **0.04** |  | 2.18 (1.01, 4.71) | **0.04** |
| Cooking |  |  |  |  | 0.30 |
| Clean fuels | **1 (Ref.)** |  |  | **1 (Ref.)** |  |
| Solid fuels | 3.30 (1.25, 8.68) | **0.015** |  | 1.73 (1.01, 2.95) | **0.04** |

**Note**: Model adjusted for age, sex, education level, marital status, smoking, drinking status, self-reported socioeconomic status, hypertension, diabetes, and stroke; Bolded *P*-values indicate statistical significance.

**Abbreviation: HR:** hazard ratio**; 95% CI:** 95% confidence interval

**Table S7. Hazard Ratios and 95% Confidence Intervals for Cognitive Frailty Related to Household Solid Fuel Use Stratified by Socioeconomic status.**

|  | **Poor (n = 3827)** | | |  | **Fair (n = 4504)** | | |  | **Good (n = 232)** | | |  |
| --- | --- | --- | --- | --- | --- | --- | --- | --- | --- | --- | --- | --- |
|  | **HR (95% CI)** |  | ***P*-value** |  | **HR (95% CI)** |  | ***P*-value** |  | **HR (95% CI)** |  | ***P*-value** | ***P* for interaction** |
| **Household fuel types** |  |  |  |  |  |  |  |  |  |  |  |  |
| Heating |  |  |  |  |  |  |  |  |  |  |  | 0.29 |
| Clean fuels | **1 (Ref.)** |  |  |  | **1 (Ref.)** |  |  |  | **1 (Ref.)** |  |  |  |
| Solid fuels | 5.66 (1.36, 23.54) |  | **0.01** |  | 1.67 (0.78, 3.57) |  | 0.18 |  | 0.24 (0.01,5.08) |  | 0.35 |  |
| Cooking |  |  |  |  |  |  |  |  |  |  |  | 0.60 |
| Clean fuels | **1 (Ref.)** |  |  |  | **1 (Ref.)** |  |  |  | **1 (Ref.)** |  |  |  |
| Solid fuels | 1.55 (0.79, 3.05) |  | 0.19 |  | 2.41 (1.19, 4.86) |  | **0.01** |  | 1.04 (0.08, 13.19) |  | 0.97 |  |

**Note**: Model adjusted for age, sex, education level, marital status, smoking, drinking status, residence, hypertension, diabetes, and stroke; Bolded *P*-values indicate statistical significance.

**Abbreviation: HR:** hazard ratio**; 95% CI:** 95% confidence interval

**Table S8. Hazard Ratios and 95% Confidence Intervals for Cognitive Frailty Related to Household Solid Fuel Use Stratified by Smoke Categories.**

|  | **Nonsmoker (n = 5243)** | |  | **Current or former smoker (n = 3320)** | |
| --- | --- | --- | --- | --- | --- |
|  | **HR (95% CI)** | ***P*-value** |  | **HR (95% CI)** | ***P*-value *P* for interaction** |
| **Household fuel types** |  |  |  |  |  |
| Heating |  |  |  |  | 0.78 |
| Clean fuels | **1 (Ref.)** |  |  | **1 (Ref.)** |  |
| Solid fuels | 2.11 (0.94, 4.71) | 0.06 |  | 2.88 (1.02, 8.13) | **0.045** |
| Cooking |  |  |  |  | 0.85 |
| Clean fuels | **1 (Ref.)** |  |  | **1 (Ref.)** |  |
| Solid fuels | 1.96 (1.03, 3.73) | **0.04** |  | 2.12 (1.04, 4.31) | **0.037** |

**Note**: Model adjusted for age, sex, education level, marital status, socioeconomic status, drinking status, residence, hypertension, diabetes, and stroke; Bolded *P*-values indicate statistical significance.

**Abbreviation: HR:** hazard ratio**; 95% CI:** 95% confidence interval

**Table S9. Hazard Ratios and 95% Confidence Intervals for Cognitive Frailty Related to Household Solid Fuel Use Stratified by Drink Categories.**

|  | **Nondrinker (n = 5229)** | |  | **Current or former drinker (n = 3334)** | |
| --- | --- | --- | --- | --- | --- |
|  | **HR (95% CI)** | ***P*-value** |  | **HR (95% CI)** | ***P*-value *P* for interaction** |
| **Household fuel types** |  |  |  |  |  |
| Heating |  |  |  |  | 0.72 |
| Clean fuels | **1 (Ref.)** |  |  | **1 (Ref.)** |  |
| Solid fuels | 2.18 (0.98, 4.83) | 0.05 |  | 2.63 (0.92, 7.53) | 0.07 |
| Cooking |  |  |  |  | 0.65 |
| Clean fuels | **1 (Ref.)** |  |  | **1 (Ref.)** |  |
| Solid fuels | 1.86 (1.02, 3.38) | **0.04** |  | 2.35 (1.06, 5.17) | **0.03** |

**Note**: Model adjusted for age, sex, education level, marital status, smoking, socioeconomic status, residence, hypertension, diabetes, and stroke; Bolded *P*-values indicate statistical significance.

**Abbreviation: HR:** hazard ratio**; 95% CI:** 95% confidence interval
